# Association of snoring and body composition in (peri-post) menopausal women

**DOI:** 10.1186/s12905-020-01025-2

**Published:** 2020-08-13

**Authors:** Yang Zhou, Fei Liu, Changbin Li, Yanwei Zheng, Jiangshan Hu, Yibei Zhou, Lulu Geng, Susu Jiang, Yincheng Teng, Minfang Tao

**Affiliations:** 1 Department of Gynecology and Obsterics, Shanghai Jiao Tong University Affiliated Sixth People’s Hospital, 600 Yishan Road, Shanghai, 200233 China; 2 Reproductive medicine center, Shanghai Jiao Tong University Affiliated Sixth People’s Hospital, 600 Yishan Road, Shanghai, 200233 China

**Keywords:** Body composition, Snoring, Menopausal transition

## Abstract

**Background:**

Little attention has been paid to whether snoring frequency is associated with body composition in menopausal women, particularly in China. This study objected to investigate the association between self-reported snoring and body composition in (peri-post) menopausal Chinese women as well as metabolic indicators.

**Methods:**

This cross-sectional study enrolled 715 participants aged 40–67 years from the Menopause Clinic in the Shanghai Sixth People’s Hospital. Participants were categorized into four subgroups stratified by self-reported snoring frequency: never, rarely (< 1 night per week), occasionally (1–2 nights per week), regularly (≥3 nights per week), while body composition was measured using bioelectrical impedance analysis (BIA). Besides, blood sample were collected to test the glycolipid indicators.

**Results:**

In our sample of investigation, regular snoring (≥3 nights per week) was found to be an independent risk factor for higher fat mass (total, upper limbs, trunk), with the highest risk of 2.4 times for fat mass of trunk after adjusting for metabolic confounders(*p* = 0.003). Meanwhile, regular snoring was independently associated with higher fat mass (total and each segment) only in menopausal transition (*p* = 0.023).

**Conclusions:**

We suggested that self-reported regular snoring may be taken as a simple alternative to predict higher fat mass (≥17.11 kg, upper quartile) in menopausal women. Similarly, body composition should be attached to the great importance to those who in menopausal transition in order to help to prevent obstructive sleep apnea (OSA).

## Background

Snoring, the manifestation of increased upper airway resistance, is commonly regarded as a reliable proxy marker of obstructive sleep apnea (OSA) [1, 2]. Moreover, regular snoring has been suggested to be correlated with obesity [3], hypertension [4] and diabetes mellitus [5]. OSA is supposed to be more prevalent in men than women, however the gap was narrowed when women approach menopause [6, 7]. Women in menopause transition are more likely to report perspective poor sleep, snoring [8], which largely affected quality life of menopausal women. In addition, previous studies have reported that menopause was an important risk factor for snoring mainly due to the declining ovarian hormones [9, 10]. Thus, it is important to combat snoring in (peri-post) menopausal women.

Meanwhile, menopause is a vital window for variations in the body composition and rising in the body weight caused by hormonal alterations [11]. However, body mass index, BMI, is not a valid measure of true obesity status in menopausal women [12]. Changes in menopause-related body composition may be covered and underestimated by stable BMI since the counteractive effect of loss of lean mass and gain of fat mass when aging. Therefore, body composition by bioelectrical impedance analysis (BIA) may be a more representative and precise instrument rather than BMI among menopausal Chinese women [13].

So far, current studies on the association of snoring and obesity have focused primarily on men and children [3, 14], while underrepresented women. In addition, any association between snoring and body composition in menopausal women has received little attention. Since it’s possible that glycolipid metabolism may confound the association, and whether snoring is associated with body composition in menopausal women independently of glycolipid metabolism confounders remains unknown. Given the evidence of the cross interplay among snoring, obesity and menopause, we aim to explore the association with snoring and body composition in menopausal women.

## Methods

### Study participants

This cross-sectional study enrolled participants who visited the Menopause Clinic in the Shanghai Sixth People’s Hospital. Han-Chinese woman aged 40–67 years passing through the menopause were recruited. Exclusion criteria were (1) with rhinitis; (2) having severe internal illnesses and/or diseases such as myocardial infarction, stroke, and cancer; (3) current smoking (at least once per week for the previous 6 months); (4) excessive alcohol drinking (at least one pack per month for the previous 6 months); (5) suffering from thyroid disease; (6) having tubercle and cachexy; (7) missing data. Ultimately,715 participants were recruited in this study.

### General questionnaire

Baseline sociodemographic information was collected from a questionnaire through face-to-face interview, which has been previously employed [8] (seen in supplementary file 1); Variables included age, marital status, employment status, education level, income per month, menopausal age, menopausal status, history of chronic disease (i.e., hypertension, diabetes mellitus, rhinitis, other diseases), besides, lifestyle (i.e., smoke, alcohol consumption) were recorded. Guiding by the Stages of Reproductive Aging Workshop (STRAW + 10) [15],participants were divided into three different menopausal subgroups, namely menopausal transition group (consecutive irregularities for over 7 days of menstrual cycle), early postmenopausal group (absence of menstrual periods for 12 months − 5 years) and late postmenopausal group (absence of menstrual periods for ≥5 years). Hypertension was defined by any prior diagnosis from the questionnaire or by the criteria recommended by the seventh report of the Joint National Committee on Prevention, Detection, Evaluation, and Treatment of High Blood Pressure (JNC7, [16]). While diabetes mellitus was identified by FPG ≥ 7 mmol/L or received any treatment for diabetes according to the WHO criteria [17].

### Snoring frequency assessment

Participants were asked by the question to assess the sleep snoring frequency, which was applied previously [18, 19]. “Over the past 4 weeks, did you snore? And if did, how many times per week?” and the options for responses were “never”, “rarely”, “occasionally”, and “regularly”, corresponding to “never”, “<1 night per week”, “1–2 nights per week”, and “≥ 3 nights per week”, respectively (seen in supplementary file 1).

### Anthropometric and lab tests

We measured and recorded participants’ weight, height. Body mass index (BMI) was computed by dividing weight in kilograms by the square of their height in meters. We took the blood pressure for all participants on the right arm three consecutive times after 5-min sitting (systolic blood pressure (SBP), diastolic blood pressure (DBP)). Blood samples were collected for the detection of serum concentration of triglyceride (TG), cholesterol (TC), high-density lipoprotein (HDL), low-density lipoprotein (LDL), and fasting blood glucose (FBG) after an overnight fast.

### Body composition

We measured the body composition by BIA (TBF-418B analyzer; TANITA) of lean mass (LM), fat mass (FM), and fat-free mass (FFM), and each segment included upper /lower limbs, and trunk. We also recorded basal metabolic rate (BMR) concurrently [20]. The well-trained staff guided the participants to take off heavy clothes, socks and shoes, and hold the hand electrodes, standing barefoot in contact with footpad electrodes [21]. Fat mass (total and each segment) and lean mass (total and each segment) were stated in the dichotomized form, with a cutoff of the highest quartile as the higher one (comparing the highest to the lower two tertiles). We defined ≥17.11 kg, ≥1.41 kg and ≥ 9.11 kg as higher total fat mass, higher fat mass of upper limbs and higher fat mass of trunk respectively.

### Statistical analyses

All statistical analyses were taken by SPSS 22.0 (IBM Corporation, Armonk, NY, USA). Data were tested for normal distribution by the Kruskal WallisH-test. Levene’s test of homogeneity of variance was also performed. Variables were presented as mean ± standard deviation (SD) when they showed normal distributions, whereas medians (inter quartile range) or values (%). One-way ANOVA (normal distributions), the Kruskal Wallis H-test (skewed continuous variables) and χ2 test (categorical variables) were carried out to compare the differences among the four groups. Snoring was analyzed as a categorical variable with never as the reference group. Relationship between body composition and snoring frequency was computed by multiple logistic regression analysis. Covariates included TG, TC, HDL, LDL, FBG, SBP, DBP, age, marital status, employment status, education level, income per month, menopausal age, menopausal status, hypertension, diabetes mellitus. Two-sided *p* < 0.05 was considered significant.

## Results

### Characteristics of the study participants based on snoring frequency

A total of 715 participants were finally entered into the study. The basic characteristics among the four groups divided by the snoring frequency (never, rarely, occasionally, regularly) were presented in Table 1. Participants were on average 51.50 ± 4.71 years of age with a mean weight of 57.45 ± 7.97 kg, and the average BMI was 22.23 ± 2.76 kg/m^2^. The mean lean mass, fat mass and fat free mass were 37.64 ± 3.10 kg, 17.52 ± 5.47 kg, and 39.95 ± 3.39 kg, respectively. Compared with non-snorers, rare and occasional snorers, regular snorers tended to be older, showed higher triglyceride, lower HDL-C, and had less income (*p* < 0.05). Moreover, there was an ascending trend in the incidence of hypertension in different snoring frequency subgroups,with 19.28% in non-snorers increasing to 40.51% in regular snorers (*p* < 0.05). However, we did not observe the difference among three menopausal status respect to the snoring frequency.

**Table 1:** Body composition and characteristics of the women distributed by snoring frequency

Variables	Snoring Frequency					*P* value
	Never	Rarely	Occasionally	Regularly	Total	
	*n* = 508	*n* = 76	*n* = 56	n = 76	*n* = 715	
Age (years)	51.37 ± 4.85	51.3 ± 4.48	52.79 ± 4.02	51.58 ± 4.41	51.50 ± 4.71	0.047
Weight (Kg)	56.68 ± 7.69	57.48 ± 8.12	58.86 ± 7.52	61.36 ± 8.73	57.45 ± 7.97	0.307
Height (cm)	160.60 ± 4.88	161.24 ± 4.46	160.46 ± 4.74	160.85 ± 4.64	160.68 ± 4.8	0.580
BMI (Kg/m^2^)	21.95 ± 2.61	22.06 ± 2.60	22.88 ± 2.92	23.71 ± 3.16	22.23 ± 2.76	0.034
BMR	1154.8 ± 109.79	1166.97 ± 121.7	1175.58 ± 99.35	1210.67 ± 120.69	1163.93 ± 112.73	0.555
TG (mmol/l)	1.07 (0.79–1.49)	1.05 (0.82–1.57)	1.18 (0.80–1.69)	1.37 (0.94–2.05)	1.11 (0.80–1.60)	0.003
TC (mmol/l)	5.21 ± 0.95	5.23 ± 1.05	5.38 ± 0.93	5.21 ± 1.07	5.23 ± 0.97	0.440
HDL-C (mmol/l)	1.61 ± 0.39	1.56 ± 0.37	1.52 ± 0.34	1.14 ± 0.14	1.55 ± 0.39	<0.001
LDL-C (mmol/l)	3.05 ± 0.76	3.01 ± 0.89	3.13 ± 0.71	3.18 ± 0.78	3.07 ± 0.77	0.192
FPG (mmol/L)	5.16 (4.82–5.52)	5.05 (4.80–5.33)	5.24 (4.75–5.55)	5.37 (4.90–5.86)	5.16 (4.81–5.53)	0.129
SBP (mmHg)	119.77 ± 15.26	122.21 ± 13.98	121.91 ± 17.85	126.19 ± 19.02	120.91 ± 15.91	0.088
DBP (mmHg)	73.29 ± 9.84	75.72 ± 8.85	75.09 ± 10.93	78.19 ± 9.8	74.23 ± 9.94	0.392
Chronic disease, n (%)						
Hypertension	97 (19.28%)	15 (19.74%)	18 (31.58%)	32 (40.51%)	162 (22.66%)	<0.001
Diabetes	16 (3.18%)	2 (2.63%)	2 (3.51%)	7 (8.86%)	27 (3.78%)	0.094
Marital status, n (%)						0.904
Married	490 (97.42%)	75 (98.68%)	56 (98.25%)	77 (97.47%)	698 (97.62%)	
Single/Separated/Divorced/Widowed	13 (2.58%)	1 (1.32%)	1 (1.75%)	2 (1.53%)	17 (2.38%)	
Menopausal status, n (%)						0.393
Perimenopause	217 (43.14%)	32 (42.11%)	20 (35.09%)	31 (39.24%)	300 (41.96%)	
Early postmenopause	189 (37.57%)	34 (44.74%)	24 (42.11%)	27 (34.18%)	274 (38.32%)	
Late postmenopause	97 (19.28%)	10 (13.16%)	13 (22.81%)	21 (26.58%)	141 (19.72%)	
Employment, n (%)	306 (60.83%)	42 (55.26%%)	29 (50.88%)	45 (56.96%)	422 (59.02%)	0.448
Education, n (%)						0.053
Junior or below	87 (17.30%)	15 (19.74%)	12 (21.05%)	19 (24.05%)	133 (18.60%)	
Senior high	160 (31.81%)	28 (36.84%)	18 (31.58%)	36 (45.57%)	242 (33.85%)	
College or above	256 (50.89%)	33 (43.42%)	27 (47.37%)	24 (30.38%)	340 (47.55%)	
Income (RMB/month), n (%)						0.011
< 1000	35 (6.96%)	7 (9.21%)	5 (8.77%)	12 (15.19%)	59 (8.25%)	
1000–3000	113 (22.47%)	23 (30.26%)	18 (31.58%)	31 (39.24%)	185 (25.87%)	
3000–5000	156 (31.01%)	18 (23.68%)	16 (28.07%)	22 (27.85%)	212 (29.65%)	
5000–10,000	131 (26.04%)	17 (22.37%)	11 (19.30%)	9 (11.39%)	168 (23.50%)	
> 10,000	68 (13.52%)	11 (14.47%)	7 (12.28%)	5 (6.33%)	91 (12.73%)	
Lean mass (kg)	37.45 ± 3.07	37.53 ± 3.13	37.95 ± 2.92	38.71 ± 3.25	37.64 ± 3.10	0.007
Upper	3.46 ± 0.42	3.45 ± 0.44	3.56 ± 0.38	3.71 ± 0.46	3.50 ± 0.43	<0.001
Trunk	21.08 ± 1.95	21.23 ± 1.94	21.22 ± 1.59	21.68 ± 2.42	21.17 ± 1.99	0.087
Lower	12.91 ± 1.36	12.86 ± 1.43	13.17 ± 1.57	13.32 ± 1.65	12.97 ± 1.42	0.063
Fat mass (kg)	16.94 ± 5.23	17.66 ± 5.18	18.58 ± 5.47	20.24 ± 6.34	17.52 ± 5.47	0.027
Upper	1.38 ± 0.53	1.44 ± 0.55	1.56 ± 0.61	1.76 ± 0.69	1.45 ± 0.57	0.002
Trunk	9.04 ± 3.33	9.56 ± 3.20	10.03 ± 3.48	10.94 ± 3.97	9.39 ± 3.46	0.044
Lower	6.55 ± 1.50	6.68 ± 1.53	7.02 ± 1.54	7.55 ± 1.90	6.71 ± 1.59	0.027
Fat-free (kg)	39.74 ± 3.34	39.83 ± 3.44	40.29 ± 3.14	41.13 ± 3.56	39.95 ± 3.39	0.695
Upper limbs	3.70 ± 0.45	3.70 ± 0.49	3.80 ± 0.42	3.97 ± 0.48	3.74 ± 0.46	0.789
Trunk	22.36 ± 2.14	22.53 ± 2.13	22.52 ± 1.70	23.05 ± 2.65	22.47 ± 2.17	0.476
Lower limbs	13.70 ± 1.47	13.63 ± 1.54	13.99 ± 1.68	14.15 ± 1.80	13.76 ± 1.54	0.645

### Body composition of the study participants distributed by snoring frequency

As presented in Table 1, compared with non-snorers, rare and occasional snorers, regular snorers had higher fat mass (upper limbs, trunk, lower limbs). In addition, we found that there was an increasing trend in the fat mass of upper limbs, trunk and lower limbs and also in lean mass of upper limbs with the increase of the sleep snoring frequency (*p* < 0.05).

### Odds ratio of snoring frequency for body composition by multiple logistic regression analysis

**Fig. 1:**
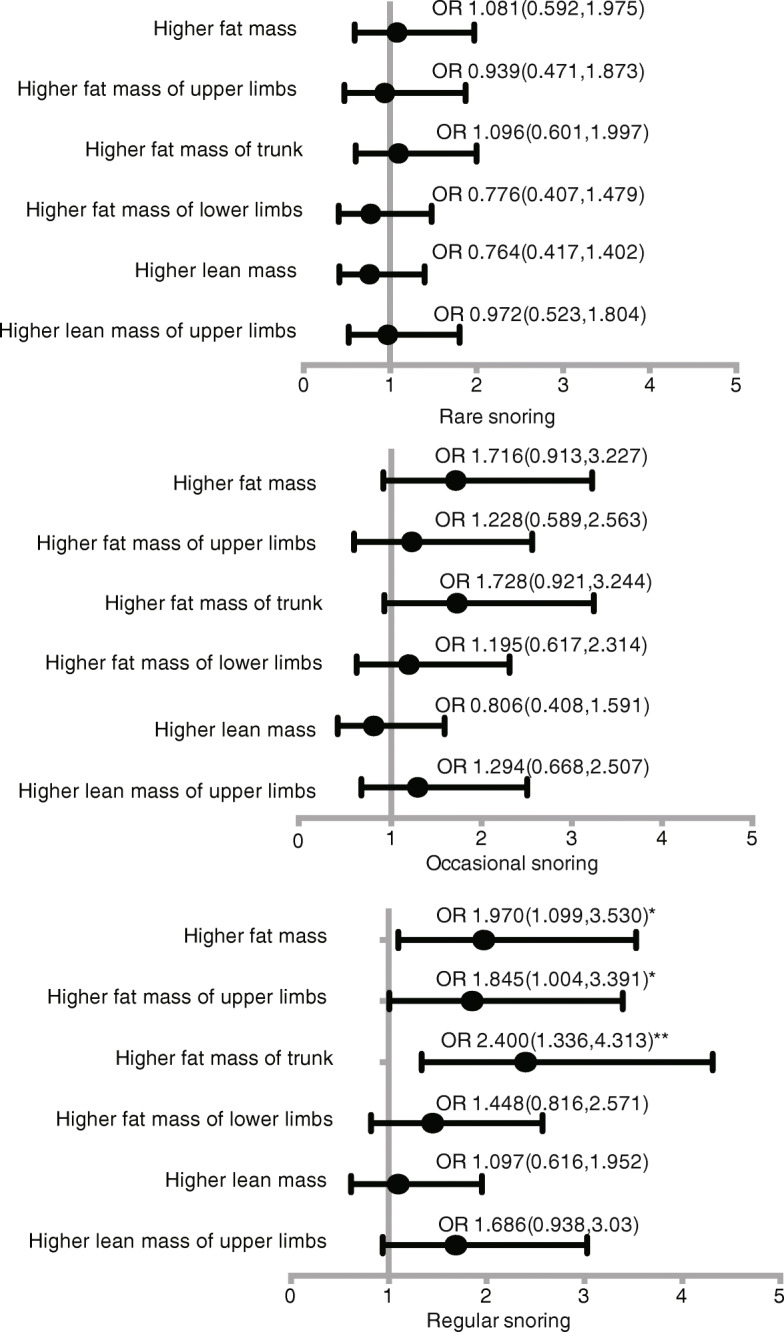
Odds ratios (95%CI) of snoring frequency for body composition in women analyzed by multivariate logistic regression. Covariates: age, BMI, TG, TC, HDL, LDL, FBG, SBP, DBP, hypertension, diabetes mellitus, menopause, income, education, employment status. *means p<0.005

We next investigated the odds ratio of snoring frequency in predicting for body composition (*p* < 0.05 in univariate analysis) after adjusting for potential confounders. As depicted in Fig. 1, compared with non, rare and occasional snoring, regular snoring was the risk predictor for higher total fat mass (≥17.11 kg) (OR = 1.970, 95%CI (1.099,3.530), *P* = 0.023), higher fat mass of upper limbs (≥1.41 kg) (OR = 1.845, 95%CI (1.004,3.391), *P* = 0.049), higher fat mass of trunk (≥9.11 kg) (OR = 2.400, 95%CI (1.336,4.313), *P* = 0.003), while other segments showed no significance after adjustments. In addition, regular snoring increased the highest odds ratio (OR) of 2.4 for fat mass of trunk among the other statistically significant body compositions.

### Independent determinants for regular snoring stratified by menopausal status

We also investigated the independent roles of body composition for predicting regular snoring in multivariate logistic regression analysis in Table 2, however, we did not observe any significance of body composition in predicting for regular snoring after adjusting confounders. Interestingly, when the participants were stratified by different menopausal status (menopausal transition, early postmenopause, late postmenopause), we observed that fat mass segments were independently associated with regular snoring in menopausal transition but not postmenopause. Total fat mass (OR = 1.134, 95%CI (1.018,1.263), *P* = 0.023), fat mass of upper limbs (OR = 3.162, 95%CI (1. 183,8.452), *P* = 0.022), fat mass of trunk (OR = 1.206, 95% CI (1.016,1.431), *P* = 0.033), fat mass of lower limbs (OR = 1.548, 95%CI (1.069,2.242), *P* = 0.021) were independent indicators for regular snoring after adjusting for confounders in menopausal transition.

**Table 2:** Odds ratio of body composition for regular snoring stratified by menopausal status by logistic regression

	Regular snoring		
	Perimenopause *n* = 300	Early postmenopause *n* = 274	Late postmenopause *n* = 141
	Odds ratio (95% CI), *P*-value	Odds ratio (95% CI), *P*-value	Odds ratio (95% CI), *P*-value
Fat mass (kg)	1.134 (1.018,1.263),0.023	0.970 (0.875,1.074),0.556	1.012 (0.876,1.170),0.867
Fat mass of upper limbs (kg)	3.162 (1.183,8.452),0.022	1.007 (0.389,2.606),0.989	0.838 (0.196,3.587),0.812
Fat mass of trunk (kg)	1.206 (1.016,1.431),0.033	0.941 (0.801,1.105),0.456	1.048 (0.834,1.316),0.689
Fat mass of lower limbs (kg)	1.548 (1.069,2.242),0.021	0.928 (0.641,1.345),0.695	0.955 (0.608,1.502),0.843

## Discussion

To our knowledge, this is the first study to document associations of snoring and body composition as well as metabolic indicators in women with regard to menopausal status. The main finding was that regular snoring (≥3 nights per week) was an independent risk factor for higher fat mass (total, trunk, upper limbs) in menopausal women after adjusting for well-established metabolic variables. Of special concern was that regular snoring had a 2.4 times significantly higher odds of higher fat mass of trunk, which was the highest among other significant body composition. This finding was in concordant with the previous study that OSA was more inclined to a central-obesity phenotype than a whole-obesity pattern [22].

Several mechanisms can interpret this association. Upper airway resistance and collapsibility caused by regular snoring could result in intermittent hypoxia and sympathetic activation, thus leading to the aggravation of obesity, especially for abdominal fat [23]. In addition, protective role of progesterone and estrogen in respiratory control vanished after menopause, which was associated with continuum from increased airway resistance (manifested as snoring) [24–26]. Taken together, menopause make women lose protective effects against snoring and further augment snore-obesity association, especially snore-central-obesity association.

Interests in obesity and OSA as regards to “which is the chicken or the egg?” has existed since the dawn of history [23, 27]. Thus, to identify the mutual effect of snoring and obesity, we also assessed the role of body composition in predicting the snoring. We found that fat mass was an independent risk factor for regular snoring only in menopausal transition not postmenopause in a multi-variable model. Taken together, we suggested that the rise in obesity may serve as a key contributor to the burgeoning prevalence of snoring in women, while menopausal transition not postmenopause period may mark this relationship.

The reason can be explained by the fact that menopausal transition is more concerned with fluctuation of sex hormone than postmenopause, which predisposed to modulate sleep regulation and breathing, thus leading to the snoring.

Besides, other independent factors for regular snoring such as higher TG, lower LDL were compatible with one study [28]. Although self-reported snoring was closely related with hypertension and diabetes. Unexpectedly, we did not find that hypertension was related with regular snoring after multiple adjustments. These divergent findings may be attributed to difference in sample size, ethnicity, culture, and the definition of hypertension and diabetes, etc. Another possible explanation is that many previous studies did not consider menopause status in to account, which may aggravate the snore-obesity association, thus overshadow the snore-hypertension/diabetes association.

However, our study should be interpreted in light of the following limitations. First, one limitation of the present study is that the cross-sectional design does not permit conclusion of causality, further prospective studies are needed to verify the association between snoring frequency and body composition. Second, self-reported snoring frequency but not polysomnography (the gold standard for diagnosing OSA), which could bring about the statistical error. However, precious study has suggest that self-report is a reliable measure [29].

## Conclusions

Regular snoring (≥ 3 times per week) may be an independent strong predictor for fat mass of trunk in menopausal women, while fat mass in turn serves as a strong predictor for regular snoring only in menopausal transition. Taken together, early detection and interventions of participant showing regular snoring and higher fat mass in menopause could have important preventive implications.

## Supplementary information

**Additional file 1.**

## Data Availability

The datasets used and/or analyzed during the current study are available from the corresponding author on reasonable request.

## Abbreviations

BIA: Bioelectrical impedance analysis
OSA: Obstructive sleep apnea
TG: Triglyceride
TC: Cholesterol
HDL: High-density lipoprotein
LDL: Low-density lipoprotein
FBG: Fasting blood glucose
BMI: Body mass index
FM: Fat mass
LM: Lean mass
FM: Fat mass
FFM: Fat-free mass
BMR: Basal metabolic rate
