# Supplementary material for: Association of snoring and body composition in (peri-post) menopausal women
Source: BMC Womens Health. 2020 Aug 13;20:175. doi: 10.1186/s12905-020-01025-2 (PMC7427281; doi:10.1186/s12905-020-01025-2)
Supplement: Supplementary file 1 — Additional file 1. [file 12905_2020_1025_MOESM1_ESM.docx]

**General information and Snoring frequency assessment**

**1. Date of birth：**_______Year_______Month_______Date

**2. Married status**

1=married; 2=unmarried；3= separated；4=divorced/widowed

**3. Education condition**

1=uneducated; 2=primary school;3=junior school;4=senior high/technical school；5=college；6=postgraduate or above

**4. Income condition (monthly/RMB)**

1＜1000；2=1000 – 3000；3=3000 – 5000；4=5000 – 10000；5≥10000

**5. Menopausal status**

Last menstrual period（LMP）：_______

Previous menstrual period (PMP): _______

Menopausal status: _______

menopausal transition group (consecutive irregularities for over 7 days of menstrual cycle)； early postmenopausal group (absence of menstrual periods for 12months -5 years)； late postmenopausal group (absence of menstrual periods for ≥ 5 years)

**6. Menopausal method judgement**

1=Natural physiological behavior；2=surgical menopause；3=chemotherapy-induced

**7. History of chronic disease**

1=Hypertension, medical history _______year; _______antihypertensive drugs

2=Diabetes, medical history_______ year; _______antidiabetic drugs

3=Rhinitis, medical history_______ year; _______ drugs

4=Tumors, medical history_______ year; _______ drugs

5=Thyroid disease, medical history_______ year; _______ drugs

6=Tubercle and cachexy, medical history_______ year; _______ drugs

7=Other severe internal illnesses (such as myocardial infarction, stroke and etc)， medical history_______ year; _______ drugs

**8. Smoking status**

Current smoking history (at least once per week for the previous 6 months)

**9. Alcohol drinking**

Excessive alcohol drinking (at least one pack per month for the previous 6 months)

**10. Snoring frequency assessment**

Over the past 4 weeks, did you snore? And if did, how many times per week?”

_______ Never: None;

_______ Rarely:＜1 night per week;

_______ Occasionally: 1–2 nights per week;

_______ Regularly: ≥ 3 nights per week
